# Premortem clinical diagnoses and postmortem autopsy findings: discrepancies in critically ill cancer patients

**DOI:** 10.1186/cc5782

**Published:** 2007-04-20

**Authors:** Stephen M Pastores, Alina Dulu, Louis Voigt, Nina Raoof, Margarita Alicea, Neil A Halpern

**Affiliations:** 1 Critical Care Medicine Service, Department of Anesthesiology and Critical Care Medicine, Memorial Sloan-Kettering Cancer Center, 1275 York Avenue, New York, NY 10021, USA

## Abstract

**Introduction:**

Limited data are available regarding the relationship of premortem clinical diagnoses and postmortem autopsy findings in cancer patients who die in an oncologic intensive care unit (ICU). The purposes of this study were to compare the premortem clinical and postmortem diagnoses of cancer patients who died in the ICU and to analyze any discrepancies between them.

**Methods:**

This is a retrospective review of medical records and autopsy reports of all cancer patients who died in a medical-surgical ICU and had an autopsy performed between 1 January 1999 and 30 September 2005 at a tertiary care cancer center. Premortem clinical diagnoses were compared with the postmortem findings. Major missed diagnoses were identified and classified, according to the Goldman criteria, into class I and class II discrepancies.

**Results:**

Of 658 deaths in the ICU during the study period, 86 (13%) autopsies were performed. Of the 86 patients, 22 (26%) had 25 major missed diagnoses, 12 (54%) patients had class I discrepancies, 7 (32%) had class II discrepancies, and 3 (14%) had both class I and class II discrepancies. Class I discrepancies were due to opportunistic infections (67%) and cardiac complications (33%), whereas class II discrepancies were due to cardiopulmonary complications (70%) and opportunistic infections (30%).

**Conclusion:**

There was a discrepancy rate of 26% between premortem clinical diagnoses and postmortem findings in cancer patients who died in a medical-surgical ICU at a tertiary care cancer center. Our findings underscore the need for enhanced surveillance, monitoring, and treatment of infections and cardiopulmonary disorders in critically ill cancer patients.

## Introduction

Major discrepancy rates between premortem clinical diagnoses and postmortem autopsy findings continue to be reported in critically ill patients admitted to the intensive care unit (ICU) [1-22]. However, there are limited data regarding the relationship of premortem diagnoses and postmortem findings specifically in cancer patients who die in an oncologic ICU [23,24]. The purposes of this study were to compare the premortem clinical and postmortem diagnoses of cancer patients who died in the ICU and to analyze any discrepancies between them.

## Materials and methods

This is a retrospective study of all patients who died in the ICU and had an autopsy performed between 1 January 1999 and 30 September 2005 at Memorial Sloan-Kettering Cancer Center, a 435-bed tertiary care cancer center in New York City. The ICU is a 'closed' 12-bed adult medical-surgical unit staffed by anesthesiology and internal medicine housestaff, critical care fellows, and full-time critical care medicine attending physicians. The ICU attending physicians conduct multidisciplinary rounds twice daily and supervise and approve all clinical decisions in collaboration with the admitting medical and surgical teams. Our standard of care includes surveillance for nosocomial infections, aggressive and early use of broad-spectrum antimicrobial agents in patients with suspected or proven infection, and routine use of antimicrobial-impregnated central venous catheters.

Consent for an autopsy is always requested from the health care proxy and/or a relative of the deceased patient by the ICU housestaff or attending physician and occasionally by the primary admitting team. Autopsies commonly are performed within 24 hours of death. The standard autopsy includes gross and histopathologic examination of all internal organs and the brain, when indicated.

All ICU admissions, deaths, and autopsies were identified by the hospital's institutional database. The following data were obtained from the electronic medical record for the ICU patients who died in the ICU and had autopsies: age, gender, admitting service (medical or surgical), underlying cancer diagnoses, and lengths of ICU stay and hospital stay. The major premortem clinical diagnoses and causes of death, including the immediate cause of death and the underlying primary disease, were recorded. The autopsy diagnoses were obtained from the final autopsy reports. Based on a review of the medical record and the autopsy diagnoses, two investigators (AD and LV) independently identified the clinical causes of death and then compared their results. If there was disagreement, the medical records were reviewed together by both investigators and a consensus on the cause of death was reached after discussion.

Discrepancies between premortem clinical and autopsy diagnoses were classified using the Goldman criteria (Table 1) [25]. For the purposes of this study, we focused only on the class I and class II major discrepancies [2]. Class I discrepancies were defined as a missed major diagnosis that, had it been made, would have changed management and might have resulted in prolonged survival. Class II discrepancies were a missed major diagnosis with no impact on treatment and survival because either the patient was already receiving appropriate therapy even though the diagnosis was not known or effective therapy was not available at the time.

**Table 1:** Goldman criteria for autopsy discrepancies [25]

Major discrepancies	Class I	Missed major diagnosis with potential adverse impact on survival and that would have changed management
	Class II	Missed major diagnosis with no potential impact on survival and that would have not changed therapy
Minor discrepancies	Class III	Missed minor diagnosis related to terminal disease but not related to the cause of death
	Class IV	Other missed minor diagnosis

Patients were categorized into three groups: (a) patients in whom a major clinical diagnosis was missed premortem (discordant cases), (b) patients in whom the premortem clinical diagnosis was confirmed on autopsy (concordant cases), and (c) patients in whom no pathologic diagnosis could be confirmed on autopsy.

### Statistical analysis

Data are presented as means ± standard deviations, absolute numbers, or percentages. Statistical analyses used included Fisher exact test and one-way analysis of variance to test for differences among the three groups. *P *values of less than 0.05 were considered significant. All statistical analyses were performed using statistical software (SPSS 12.0; SPSS Inc., Chicago, IL, USA). The study was approved by the institutional review board, which waived the need for informed consent.

## Results

Between 1 January 1999 and 30 September 2005, 658 (20.2%) of the 3,257 patients admitted to the ICU died. Of the 658 deaths, 86 (13%) had an autopsy. During the study period, our autopsy rates averaged 13% per year (range, 7.7% to 21.2% per year).

Of the 86 patients who underwent an autopsy, 38 (44%) were women and 48 (56%) were men. The mean age was 54 ± 16 years. The mean length of stay (LOS) in the ICU was 9 ± 8 days, and the mean LOS in the hospital was 19 ± 18 days. Twenty-four patients (28%) were surgical patients and 62 (72%) were medical. Of the 24 surgical patients, 10 (42%) underwent thoracotomy for lung or esophageal cancer, 10 (42%) gastrointestinal/hepatobiliary surgery for hepatic or pancreatic cancer, 2 (8%) orthopedic surgery for sarcoma, 1 head and neck cancer surgery, and 1 gynecologic cancer surgery. Of the 62 medical patients, 25 (40%) had undergone hematopoietic stem cell transplantation (HSCT), 18 (29%) had hematologic malignancies (leukemias or lymphomas), and 19 (31%) had solid tumors.

Major missed diagnoses (discordant cases) were noted in 22 patients (26%) (group 1): 12 (54%) patients had class I discrepancies, 7 (32%) had class II discrepancies, and 3 (14%) had both class I and class II discrepancies. Among the 22 discordant cases, 6 had undergone surgery, 6 had hematologic malignancies, 6 had solid tumors, and 4 underwent HSCT.

Opportunistic infections were the most common class I discrepancies, followed by cardiac complications (thrombotic endocarditis, myocardial infarction, and heart failure) (Table 2). The opportunistic infections were due to one of several pathogens (viral, fungal, bacterial, and parasitic). The lung was the most commonly infected site, with pneumonia and empyema present in seven patients, followed by central nervous system infections (two patients), gastrointestinal infections (two patients), and widely disseminated disease (two patients). The majority of class II discrepancies were accounted for by cardiopulmonary complications (*n *= 7) attributed to pulmonary emboli and thrombotic endocarditis (Table 2).

**Table 2:** Class I and class II discrepancies

			***N***
	Opportunistic	VRE pneumonia	2
	infections	Legionella pneumonia	1
	(*n *= 10)	PCP pneumonia	1
		Invasive aspergillosis	1
		Candida empyema	1
Class I		VZV meningoencephalitis	1
discrepancies		HSV esophagitis	1
(*n *= 15)		CMV pneumonia	1
		Disseminated necrotizing toxoplasmosis	1
	
	Cardiac	Ischemic cardiomyopathy	2
	complications	Thrombotic endocarditis	2
	(*n *= 5)		
		Congestive heart failure	1

	Cardiopulmonary	Pulmonary embolism	4
Class II	complications		
discrepancies	(*n *= 7)	Thrombotic endocarditis	2
(*n *= 10)		Pulmonary hemorrhage	1
	
	Opportunistic	Candidemia	1
	infections (*n *= 3)	VRE meningitis	1
		CMV proctitis	1

CMV, cytomegalovirus; HSV, herpes simplex virus; PCP, pneumocystis carinii pneumonia; VRE, vancomycin-resistant enterococcus; VZV, varicella-zoster virus.

Clinical diagnoses were confirmed by autopsy in 49 patients (57%) (group 2). Most of the confirmed diagnoses were due to bacterial or fungal infections. Autopsy was inconclusive in 15 patients (17%) (group 3). Of the 15 patients, 12 (80%) were medical patients and 3 (20%) were surgical. The majority of group 3 patients died of multiple organ failure and systemic inflammatory response of unknown etiology, and no specific cause of death could be discerned on autopsy. The autopsies of these patients showed diffuse alveolar damage in the lung and diffuse non-specific inflammatory response with scaring and fibrosis in other organs, and positive cultures were not obtained.

There were no statistically significant differences in age or gender between the patients who had missed major diagnoses (group 1) and those with autopsy confirmation of premortem clinical diagnoses (group 2) (Table 3). However, the patients with no pathologic diagnosis made on autopsy (group 3) had a significantly longer ICU LOS compared to those with autopsy confirmation of premortem clinical diagnoses (*p *= 0.05). Overall, patients with autopsy confirmation of premortem clinical diagnoses were not significantly different from those with missed diagnoses (*p *= 0.11).

**Table 3:** Characteristics of critically ill cancer patients who underwent autopsy

	Missed major diagnosis (*n *= 22)	Clinical diagnosis confirmed (*n *= 49)	No pathologic diagnosis made (*n *= 15)	*P *value
Gender (males/females)	12:10	29:20	7:8	0.688
Age (years)	59 ± 12	55 ± 15	50 ± 19	0.223
ICU LOS (days)	7.5 ± 7	8 ± 10	15 ± 14	0.039
Hospital LOS (days)	15 ± 13	18 ± 16	29 ± 26	0.054

Data are presented as means ± standard deviations, absolute numbers, or percentages. ICU, intensive care unit; LOS, length of stay.

## Discussion

In this study, we found an overall discrepancy rate of 26% between the premortem clinical and autopsy diagnoses in cancer patients who died in a medical-surgical ICU at a tertiary cancer center. Our discrepancy rate of 26% is within the range of discrepancy rates (5% to 32%) that have been reported for autopsies performed in the general adult ICU population [1-22]. To our knowledge, however, only two previous autopsy studies have examined diagnostic discrepancy rates in cancer patients who died in the ICU [23,24]. Gerain and colleagues [23] reported a 59% major discrepancy rate in a medical oncologic ICU population. Unlike in our findings, the majority of major discrepancies were due to complications of the cancer itself or its treatment (for example, non-cardiogenic pulmonary edema, acute hemorrhage, and pulmonary embolism) rather than infection. We ascribe the marked difference in the discrepancy rates between the study by Gerain and colleagues [23] and our study (59% versus 26%) to the type of cancer patient population studied (medical versus mixed medical-surgical) and to improved diagnostic techniques and therapeutic strategies in recent years. However, when we compare our findings in a select patient population (the HSCT subgroup) to a similar HSCT population study [24], we observed an almost comparable, low discrepancy rate (16% versus 7%).

Opportunistic infections accounted for the majority (67%) of class I discrepancies. In contrast to previous studies that showed a predominance of fungal infections in immunocompromised patients [1,23], the opportunistic infections in our study were represented by various pathogens (viral, fungal, and parasitic) (Table 2). We ascribe these findings to the increasing exposure of our patients to broad-spectrum antimicrobials that effect the terminal flora and promote the emergence of more virulent and resistant nosocomial infections [26,27]. Our findings reinforce the difficulty of diagnosing different infectious entities such as nosocomial pneumonia and fungal and viral infections in critically ill patients [23,24]. We suggest that novel microbiologic identification with non-culture techniques, including serologic tests, immunohistologic methods, polymerase chain reaction, and molecular-probing technologies, be introduced to aid in the rapid diagnosis of these virulent infections [28,29].

In this study, we describe a category of patients (group 3, *n *= 15) who experienced prolonged ICU and hospital LOSs and had uncertain premortem diagnoses, and their autopsies were inconclusive, showing only non-specific, chronic inflammatory, and fibrotic changes in various organs, including the lung, kidney, and liver. These findings are not unexpected as it is well known that autopsies of patients who die after a prolonged period of resuscitation and support in the ICU typically report multiple organ failure as the primary cause of death regardless of the different primary diagnoses [30]. Thus, in our opinion, postmortem information may be similarly limited in providing a specific diagnosis of the cause of death in cancer patients who die after a prolonged ICU and hospital LOS (Table 3).

The 13% average yearly autopsy rate in our study is much lower than that of other published postmortem studies from adult ICUs [1-6,8,11-17]. We ascribe our lower ICU autopsy rate to one of three possibilities. First, in our center, the physician caring for the patient during hospitalization may differ from the outpatient physician who has a long-standing rapport with the family. Additionally, when the patient is admitted to the ICU, the critical care team assumes primary care. Thus, there may not be a single physician with a close enough relationship to the patient's next of kin at the time of death to obtain consent for an autopsy. Second, due to the frequent use of advanced high-tech investigative modalities available at our center, both physicians and family members may perceive that the autopsy will have a low yield. Third, when patients with advanced cancer die, physicians and family members often attribute the death to the expected complications of the malignancy. In this circumstance, it is perceived that an autopsy is unnecessary.

Our study has several limitations, including the retrospective study design and selection bias that may have occurred; physicians and family members of patients with premortem diagnostic uncertainty would have been more likely to pursue an autopsy than in cases in which all parties were certain of the diagnoses and the outcome was predictable. Similar to prior studies [13,15], we were unable to fully account for all the premortem diagnostic investigations that were performed on all the autopsied patients. Nevertheless, we believe that our findings may be extrapolated to similar critically ill cancer patients treated in general ICUs.

## Conclusion

Our study suggests that missed major diagnoses with potential impact on treatment and survival were noted in 26% of critically ill cancer patients admitted to an oncologic ICU. The missed major diagnoses were commonly due to opportunistic infections and cardiac complications. Our findings underscore the need for enhanced premorbid surveillance, monitoring, and treatment of infections and cardiopulmonary disorders in critically ill cancer patients. However, given the limitations of present-day microbiologic evaluation and treatment and cardiac imaging at the ICU bedside, the autopsy remains an invaluable tool for retrospective diagnostic understanding of difficult cases, medical education, and quality assurance.

## Key messages

• Missed major diagnoses with potential impact on treatment and survival were noted in 26% of cancer patients admitted to an oncologic ICU.

• Opportunistic infections and cardiac complications were the most commonly missed major diagnoses.

• Our findings underscore the need for enhanced surveillance, monitoring, and treatment of infections and cardiopulmonary disorders in critically ill cancer patients.

## Abbreviations

HSCT = hematopoietic stem cell transplantation; ICU = intensive care unit; LOS = length of stay.

## Competing interests

The authors declare that they have no competing interests.

## Authors' contributions

SMP, AD, and LV were responsible for the study design and data analysis. All authors were involved in drafting the manuscript and have full access to the data and take full responsibility for its integrity. All authors read and approved the final manuscript.
